# Trauma-related disorders and the bodily self: current perspectives and future directions

**DOI:** 10.3389/fpsyg.2023.1166127

**Published:** 2023-05-19

**Authors:** Daniela Laricchiuta, Carlo Garofalo, Claudia Mazzeschi

**Affiliations:** Department of Philosophy, Social Sciences and Education, University of Perugia, Perugia, Italy

**Keywords:** trauma, post-traumatic stress disorder, externalizing behaviors, aggression, body-oriented therapy, sensorimotor therapy

## Abstract

Trauma-related disorders are debilitating psychiatric conditions that influence people who have directly or indirectly witnessed adversities. Dramatic brain/body transformations and altered person's relationship with self, others, and the world occur when experiencing multiple types of traumas. In turn, these unfortunate modifications may contribute to predisposition to trauma-related vulnerability conditions, such as externalizing (aggression, delinquency, and conduct disorders) problems. This mini-review analyzes the relations between traumatic experiences (encoded as implicit and embodied procedural memories) and bodily self, sense of safety for the own body, and relationship with others, also in the presence of externalizing conducts. Furthermore, an emerging research area is also considered, highlighting principles and techniques of body-oriented and sensorimotor therapies designed to remodel bodily self-aspects in the presence of trauma, discussing their potential application with individuals showing externalizing problems.

## 1. Introduction

Embodied cognition perspective suggests that high-order mental processes are based on low-order integration of signals associated with visual, auditory, vestibular, visceral, somatosensory, and motor functions (Fernandino and Iacoboni, 2010). Low-level (bottom-up) sensorimotor processes and high-level (top-down) representations of the body combine with each other, leading to an inclusive and ongoing bodily domain (Balconi, 2010), involving body ownership and a sense of self (Gallagher, 2000; Tsakiris, 2010). Thus, the self is rooted in the body. Starting from the body borders that differentiate between inside and outside (Ogden, 2018), bodily self-consciousness encompasses self-location, self-identification with the body, and the first-person perspective (Aspell et al., 2012). All these aspects are interconnected to create a sense of self that is embodied, supporting the sense of agency over one's own body and over the space surrounding the body (peripersonal space, PPS) (Rizzolatti et al., 1997; Brozzoli et al., 2012; Serino et al., 2013; Salomon et al., 2017). Moreover, it is important also to consider interoceptive bodily awareness, defined as the representation of the body's internal state (Cameron, 2001; Craig, 2003; Farb et al., 2015).

Traumatic events, especially at an early age and in an interpersonal context, may deteriorate the experience of the body as a safe entity (Van der Kolk, 2014; Laricchiuta et al., 2023) in all domains. Early trauma, involving sexual, physical, or emotional abuse, and/or neglect, may therefore disrupt the orientation toward the body (Bernstein et al., 2003; He et al., 2019). In some cases, sensations of comfort and trust in bodily experiences may be substituted by bodily perceptions associated with the implicit memory of the trauma. In accordance with the dual representation theory (Brewin, 2011), explicit and implicit memories are crucial for storing traumatic information. Explicit memory refers to semantic, episodic, and autobiographic memory that can be verbally accessed. Implicit memory cannot be retrieved, and therefore, is unconscious. Such a memory typically involves bottom-up somatosensory manifestations and intense re-experiencing of somatic sensations of the traumatic event (Hellawell and Brewin, 2004). Van der Kolk (2014) articulated that the “somatic memory” of trauma may conduct to estrangement from own body, with bodily signals perceived as alarming. Thus, the somatic experiencing approach to trauma focuses on the hypothesis that trauma is “blocked” in the body (Van der Kolk, 1994; Levine, 1997). All individuals are physiologically programmed to flee, fight, or freeze in the face of adversity. If the natural responses to danger are impeded, the unfinished defensive actions become blocked in the body, even long after the event has passed. Continued mobilizing (fight or flight responses) or immobilizing (freeze response) defenses are reflected by physiologic states of autonomic hyper- and hypo-arousal, respectively, very often considered as the hallmark symptoms of trauma (Siegel, 1999). Inflexibility between the defensive systems and their expression in the absence of danger involves chronic dysregulated arousal, contributes to maintain the traumatization, and allows to put into action externalizing and internalizing post-traumatic conducts (Panuccio et al., 2022). Internalizing problems feature mood or emotion as their primary characteristics including symptoms such as anxiety, depression, anhedonia, and withdrawal. Externalizing problems include aggressiveness, delinquency, oppositional defiant disorder, and conduct disorder (Achenbach et al., 1991; Kovacs and Devlin, 1998; Laricchiuta et al., 2023).

What is being reported so far consents to re-think at post-traumatic stress disorder (PTSD). This pervasive pathological condition includes re-experiencing, avoidance, hyperarousal symptoms, and negative alterations in mood and cognition (APA, 2013). At the level of defensive actions, it is mainly characterized by bodily manifestations of fight-and-flight defensive systems. PTSD symptomatology implies an association between high-level and low-level processes, namely, the bottom-up multisensory mechanism is mainly involved during re-experiencing and hyperarousal symptoms, in which the trauma is relived as if it was re-occurring at the present moment, with concurrent bodily reactions. Conversely, avoidance symptoms have been associated with a top-down over-modulation of emotional reaction, characterized by emotional detachment/restricted affect coping style (Frewen et al., 2008, 2012; Lanius, 2010), which has also been posited to underlie the development and manifestation of callous and aggressive tendencies (Kosson et al., 2018).

In this framework, trauma-related disorders can alter the representation of, and relation with, the self at multiple levels, including cognitive, bodily, and social levels. As theories proliferate and research accumulates in the area, a concise yet comprehensive review can represent useful-to-go resources for scholars and practitioners working in the field and can facilitate developments of research agendas across disciplines. In the next sections, we attempted to synthesize the largely separate literatures on the impact of traumatic experiences on the bodily self as construed in the foregoing, also integrating the sparse investigations into externalizing syndromes. Finally, body-oriented and sensorimotor therapies, designed to remodel bodily self-aspects, are proposed as emerging care approaches in the presence of traumatization and externalizing problems.

## 2. Post-traumatic repercussions on bodily self

In the presence of traumatization, the subjective perception and experience of the body may be catastrophically and fearfully oriented (Sullivan et al., 1995). Catastrophic and fearful orientation refers to an amplified negative perception of bodily signals associated with the tendency to ruminate upon, magnify, and feel helpless when facing adverse bodily sensations (McNally, 2002). Body vigilance is high in order to monitor interoceptive bodily signals and perturbations in search of illness signs (Zvolensky and Forsyth, 2002; Pieritz et al., 2015; Tsur et al., 2017, 2018). This tendency increases psychosomatic symptoms (Lamela and Figueiredo, 2013) and body shame (Talmon and Ginzburg, 2018).

Nevertheless, post-traumatic catastrophic orientation to bodily signals may not be the only long-term and self-related psychopathological reaction to trauma. Traumatized children may exhibit a developmental trauma disorder, which includes multifaceted biopsychosocial responses to attachment disruption (D'Andrea et al., 2012; Ford et al., 2018). Furthermore, during adulthood, a complex PTSD (CPTSD) may transpire (van der Kolk et al., 2005), characterized by the abolishment of self-organization, negative self-concept, and affective dysregulation capacities (Cloitre et al., 2018). Tsur (2020) reported that prolonged experiences of interpersonal trauma during childhood were associated with a lack of trust in one's own body. Based on these findings, they suggested that the reaction to trauma that characterizes CPTSD—rather than the exposure to trauma *per se—*is consequential for one's orientation to the body.

Very often, traumatized individuals with CPTSD respond to potential or experienced danger with dissociation, which may have a negative impact on the wellbeing of their bodies (Haven and Pearlman, 2004; Haven, 2009). Intrapsychic functions of dissociation—which refers here to barring some aspects of mental functioning from conscious awareness—may reflect the need not to feel, know, be oneself, and mismanage the threat that connection poses. In turn, all these needs reflect the necessity to separate oneself from intolerable effects, traumatic memories and knowledge, unacceptable aspects of oneself, and dangerous interpersonal relationships. Critically, the dissociative subtype of PTSD (PTSD + DS) is characterized by depersonalization (feeling parts of the body or the entire body as detached and out of control) and derealization (feeling external surroundings as unreal, dreamlike, or distorted) (Lanius et al., 2012; Spiegel et al., 2013).

Interestingly, Rabellino et al. (2018) explored the rubber hand illusion (RHI), an experimental paradigm utilized to manipulate the sense of body ownership through a temporary illusion (Ehrsson et al., 2004), in subjects with PTSD and PTSD + DS, as compared to healthy controls. The illusion effect was lower in the subjects with PTSD and more variable in subjects with PTSD + DS, as compared to the controls. These findings indicate that subjects with PTSD may have a rigid body representation as an avoidance strategy, with top-down cognitive processes that weaken the impact of manipulation of body ownership. Conversely, the response elicited in PTSD + DS subjects appeared to be related to an increased vulnerability to manipulation of embodiment, which in turn associated with a disruption of multisensory integration processes. In view of the neurocognitive model, the mechanisms of embodiment and its manipulation are related to feelings of owning and controlling the body that arise as an interaction between the current multisensory input and internal models of the body (Tsakiris, 2010; Ratcliffe and Newport, 2017). Accordingly, neuroimaging studies of subjects with PTSD + DS have suggested altered activity in brain regions involved in multisensory integration (Simeon et al., 2000; Lanius et al., 2002; Felmingham et al., 2008), such as modified vestibular nuclei functional connectivity with key cortical vestibular regions (Harricharan et al., 2017) and changed functional connectivity of the left flocculus (the cerebellar component of the vestibular system) with key regions of the default mode network (the cortical component of the attentional system) (Rabellino et al., 2022).

Thus, significant disruption of multisensory integration processes manifests as either a hyper-rigid or extremely weak representation of the body that is critical for understanding the relationship with bodily self and surroundings. Depersonalization and derealization prevent the individual from creating a stable space as a defensive zone around the body, corresponding to an unstable self-other distinction (Rabellino et al., 2020).

## 3. Implications for externalizing disorders

Some of the consequences of trauma described above—such as shame related to the body and the self as well as affective dysregulation—are robust correlates of externalizing behavior in community, clinical, and correctional samples (Velotti et al., 2014, 2017; Garofalo et al., 2018). This stresses the importance of increased attention to the body and mind/body relations in individuals at risk for externalizing behavior. One reason why attention to these matters in offending populations has been limited may be due to the fact that a portion—and perhaps the most severe—of this population does not apparently show typical signs of bodily alterations. Therefore, scholars and practitioners may operate under the assumption that these individuals have either not been exposed to early traumatic experiences or have been resilient against them. However, few but convincing evidence suggests that this may be far from the truth. First, because of the direct evidence that offenders report, on average, higher levels of traumatic experiences across the lifespan compared to non-offenders (Wolff et al., 2009; Adshead and Ferrito, 2015; Gueta et al., 2021; for a recent review, see Pettus, 2023). Second, in offenders with personality disorders, early, versatile, and chronic antisocial tendencies have been associated with reduced interoceptive awareness (Nentjes et al., 2013), suggesting that the expression of antisocial traits and behaviors may be influenced by an attenuated sensitivity to one's own bodily signal. The most severe forms of externalizing disorders in adulthood (e.g., psychopathy; DeLisi, 2009; Gillespie et al., 2023) can also be associated with hyposensitivity to threat and reduced ability to detect sources of threat (Hoppenbrouwers et al., 2016; Fanti et al., 2020; Driessen et al., 2021). At the same time, these individuals are also prone to interpret ambiguous situations as threatening and over-react activating fight responses in the form of aggressive behavior (Smeijers et al., 2017, 2019). Notably, findings concerning interoceptive awareness and threat detection are consistent—albeit indirectly—with a pattern of previous or current victimization.

Third, research suggests that violent offenders possess an enlarged perception of their PPS coupled with a hyper-sensitivity and an exaggerated neural response to perceptions of personal space invasion, a pattern that has been attributed to a tendency to hostile interpretation bias (Schienle et al., 2017) that is also consistent with traumatization. Fourth, recent findings of specific bodily dysfunctions linked reduced pain distress to externalizing (i.e., impulsive and irresponsible) traits and also to reduced estimation of others' pain distress, which is a purported causal antecedent of aggressive behavior (Brazil et al., 2022).

It is noteworthy that calls to place more emphasis on the assessment and therapy of mind/body relations have been historically produced in the criminology literature although these calls have often fallen in a vacuum. For instance, Ferrell (1999) argued that “Perhaps the most critical of situations, the most intimate of cultural spaces in which crime and crime control intersect are those in and around the physical and emotional self” (p. 413). Accordingly, Ferrell advocated for a “criminology of the skin” that could explain criminal behavior investigating embodied and affective meanings of crime for the self. From this perspective, externalizing behaviors can be interpreted as attempts to fill an emptied bodily self with experiences that have a strong physiological resonance (e.g., Lyng, 2004). Arguments consistent with a focus on the body and the self-body relations have been reinvigorated by Vaughn and DeLisi (2018)'s criminal energetics theory of criminal enhancement and attenuation, which suggests that several key tenets of criminal career paradigms can be accounted for by focusing on indices pertaining to the body and the individual's experience of the body. Taken together, these empirical and theoretical perspectives align with a broad understanding that the self and the body have a crucial yet often unattended relevance in the understanding of externalizing conduct.

## 4. Body-oriented interventions for traumatized individuals: extension to externalizing disorders

Therapists who work with traumatized individuals recognize that trauma-related disorders have extreme complexity. Traumatized subjects do not just suffer memories of distressing events, but they show bodily responses to dysregulated emotions (Ogden et al., 2006).

Traditional “talk-therapy” approaches tend to address the explicit and declarative components of trauma. In these approaches, it is presupposed that change occurs in a top-down direction. However, as the explicit memory is recalled, the somatosensory traces of the trauma are simultaneously activated, frequently leading to a re-experiencing of somatoform symptoms that can include autonomic dysregulation, dissociative defenses associated with hyper- and hypo-arousal states, intrusive sensory experiences, and involuntary movements (Aposhyan, 2004). The body sensations are interpreted as current rather than past data. In turn, the intensity of trauma-related emotions and sensorimotor reactions disorganizes the cognitive capacities for top-down regulation (LeDoux, 2002; Schore, 2002).

Thus, a different approach to treatment may be helpful: adding bottom-up approaches to top-down therapy. In sensorimotor psychotherapy, bodily experience becomes the primary entry point of therapy, attending to the patient's body directly and working on implicit memories in order to modify procedural learning and dysregulated autonomic arousal (Ogden and Minton, 2000; Ogden et al., 2006). Traumatized patients are helped to rediscover their unfinished defensive actions via tracking their bodily movements and sensations. Somatic bottom-up interventions that address the repetitive, unbidden, physical sensations of hyper- or hypo-arousal can then be integrated with more traditional top-down interventions that help to transform the narrative of the trauma and facilitate the development of a reorganized somatic sense of self.

Recently, Classen et al. (2021) provided support for sensorimotor psychotherapy, a powerful body-oriented approach aimed to address chronic fear states in the body related to complex trauma, namely, significant improvements were found in awareness of somatic experience, anxiety, and soothing receptivity when comparing body-oriented treated women to no-treated women. The improvements resembled to shift from experiencing the body as a source of hurt to a place of healing.

In the same line, the broad concept of body awareness has been described as a key element and a mechanism of action for other mind/body approaches, such as mindfulness-based therapies and basic body awareness therapy (Mehling et al., 2011). In a safe setting, these approaches permit the subject to progressively shift the attentional focus away from thinking about threatening the body toward immediately feeling body sensations (e.g., muscular effort, joint activity, breathing, heart rate acceleration, and without judgments) to further reduce rumination and arousal (Farb et al., 2015). Mehling et al. (2018) developed an integrative exercise program for PTSD veterans that combined aerobic and resistance exercises with yoga movements and postures as well as mindfulness-based principles. This mindfulness training may support decoupling the usual reactions to unpleasant experiences and thoughts, possibly increasing the ability to downregulate hyperarousal (Kearney et al., 2012; Stephenson et al., 2017). Participants in the integrative exercise group gave high ratings for feasibility and acceptability and demonstrated greater improvement in PTSD symptom severity compared with a control group.

Finally, physical therapists from 13 countries working with the basic body awareness therapy method in mental health care were interviewed in six focus groups about what effects they have experienced in their work with patients, reporting that the therapy mainly worked by helping the patients to be in better contact with their bodily self (Gyllensten et al., 2019).

Although we are not aware of attempts to apply sensorimotor psychotherapy to clients with externalizing disorders such as offenders in correctional or forensic psychiatric hospitals, several studies from different countries have provided preliminary testing of the feasibility, acceptability, and effectiveness of mindfulness-based interventions for youth and adults in correctional settings, with a special emphasis on reducing aggression (Fix and Fix, 2013; Barrett, 2017; Simpson et al., 2018; Bouw et al., 2019; Davies et al., 2021). By and large, these approaches were well-received by staff and patients, but there was no convincing evidence that these interventions promoted clinically meaningful improvements in patients' wellbeing and reductions in maladaptive behavior compared to treatment as usual. Crucially, the scientific rigor of most studies on the topic was severely hampered by methodological and practical limitations that make generalization of the preliminary promising results challenging (Fix and Fix, 2013; Simpson et al., 2018). Importantly, preliminary findings based on more robust investigations of interventions that make use of mind/body approaches with these populations show promise. For instance, prisoners who participated in a 10-week yoga course—compared to a wait list control group—showed improvements in positive affect, perceived stress, and psychological symptoms, as well as improvements in cognitive and behavioral control in a “Go/No-Go Task” (Bilderbeck et al., 2013). Based on a systematic review, scholars have suggested that the positive effects of these kinds of mind/body interventions could be due to alterations in gene expression that contribute to a reduced risk of inflammatory reactions to stress (Buric et al., 2017).

The approaches proposed here for individuals with trauma-related externalizing disorders are also consistent with promising results obtained with trauma-informed treatments in justice-involved youth with histories of trauma (for a review, see Zettler, 2021) and could be combined with such treatments to enhance effectiveness. Specifically, trauma-informed adaptations of several therapeutic approaches (e.g., cognitive behavioral therapy, family functional therapy, and aggression replacement training) as well as trauma-informed programs specifically developed for justice-involved youth [e.g., skills-based programs, trauma affect regulation guide for education and therapy (TARGET), think trauma training, and sanctuary model] have been proven effective in reducing behavioral infractions and institutional violence although evidence of positive effects in the longer term (e.g., recidivism or post-release adjustment) are scarce. Of note, these treatments all share a focus on integrating physiological and psychological processes disrupted by traumatic experiences, hence lending themselves to a more explicit integration of sensorimotor techniques.

In line with the increased attention to this kind of approach to reduce externalizing behavior, Pettus (2023) has cogently advocated the implementation of trauma-responsive approaches to re-entry after imprisonment; we argue that such approaches should rely (also) on mind/body interventions delivered before re-entry as they can intervene in the aftermath of trauma-related externalizing psychopathology and in turn reduce recidivism risk.

## 5. Conclusion

In this mini-review, we have integrated the largely separate literature on trauma, trauma-related disorders, and bodily and mind/body dysfunctions, extending to the often-neglected area of externalizing psychopathology and closing with future treatment perspectives. In doing so, we have supported the relevance of mind/body interventions to address the multifarious negative consequences of traumatic experiences that crystallize over time and generate immense suffering to trauma survivors and—especially in the case of externalizing behavior and offending—to their environment as well. These interventions hold promise to be effective trans-diagnostic approaches addressing the traumatic roots of diverse symptomatic presentations. We hope this study will inspire future multidisciplinary research into basic mechanisms that connect traumatic experiences and mind-body alterations, externalizing behavior to further optimism that tailor interventions offered more timely and with more precision.

## Author contributions

DL conceptualized the article and performed the first literature data curation. CM acquired the funding to administer the project. All authors wrote the original draft, reviewed, and edited the manuscript. All authors contributed to the article and approved the submitted version.
